# B cell activation via immunometabolism in systemic lupus erythematosus

**DOI:** 10.3389/fimmu.2023.1155421

**Published:** 2023-05-15

**Authors:** Shigeru Iwata, Maiko Hajime Sumikawa, Yoshiya Tanaka

**Affiliations:** ^1^ Department of Rheumatology and Clinical Immunology, Wakayama Medical University, Wakayama, Japan; ^2^ First Department of Internal Medicine, University of Occupational and Environmental Health, Kitakyushu, Japan

**Keywords:** SLE, B-cell, immunometabolism, glutaminolysis, mitochondria

## Abstract

Systemic lupus erythematosus (SLE) is an inflammatory autoimmune disease involving multiple organs in which B cells perform important functions such as antibody and cytokine production and antigen presentation. B cells are activated and differentiated by the primary B cell receptor, co-stimulatory molecule signals—such as CD40/CD40L—, the Toll-like receptors 7,9, and various cytokine signals. The importance of immunometabolism in the activation, differentiation, and exerting functions of B cells and other immune cells has been widely reported in recent years. However, the regulatory mechanism of immunometabolism in B cells and its involvement in SLE pathogenesis remain elusive. Similarly, the importance of the PI3K-Akt-mTOR signaling pathway, glycolytic system, and oxidative phosphorylation has been demonstrated in the mechanisms of B cell immunometabolic activation, mainly in mouse studies. However, the activation of the mTOR pathway in B cells in patients with SLE, the induction of plasmablast differentiation through metabolic and transcription factor regulation by mTOR, and the involvement of this phenomenon in SLE pathogenesis are unclear. In our studies using activated B cells derived from healthy donors and from patients with SLE, we observed that methionine, an essential amino acid, is important for mTORC1 activation. Further, we observed that splenic tyrosine kinase and mTORC1 activation synergistically induce EZH2 expression and plasmablasts by suppressing BACH2 expression through epigenomic modification. Additionally, we identified another mechanism by which the glutaminolysis-induced enhancement of mitochondrial function promotes plasmablast differentiation in SLE. In this review, we focused on the SLE exacerbation mechanisms related to the activation of immune cells—especially B cells—and immunometabolism and reported the latest findings in the field.

## Introduction

B cells play a crucial role in systemic lupus erythematosus (SLE) pathogenesis (1–3). Nonspecific therapies such as glucocorticoids and immunosuppressive drug administration are indicated for SLE. In recent years, the efficacies of hydroxychloroquine (HCQ), mycophenolate mofetil (MMF), anti-BAFF antibody belimumab, and anti-IFN-α receptor antibody anifrolumab have been confirmed, and groundbreaking therapeutic advances have been made (4). Additionally, practical and achievable disease assessment indices, such as lupus low disease activity state (LLDAS), and definitions of remission in SLE (DORIS) criteria have been proposed. In contrast, treat-to-target strategies are being developed (5). However, many cases are refractory to treatment, so further elucidation of the pathogenesis mechanisms and research on novel therapeutic targets are essential to reduce organ damage progression, improve patient quality of life (QOL), and improve the long-term prognosis. In this review, we focused on the latest findings associated with the activation mechanism of B cells via immunometabolism and their involvement in SLE pathogenesis.

## Reported mechanisms of aberrant B-cell activation in patients with SLE

B cells play an important role in autoimmune diseases by performing functions such as antibody production, cytokine production, and antigen presentation. Under normal conditions, a self-tolerance mechanism operates during B-cell differentiation to eliminate or inactivate self-reactive B-cell receptors (BCRs) that recognize self-antigens. Self-tolerance mechanisms include (i) clonal loss, (ii) receptor editing, (iii) anergy, and (iv) lack of co-stimulation. Regulatory cells with (i) and (ii) mainly occur in the central system. In contrast, those with (iii) and (iv) mainly occur in the peripheral system (6). However, in autoimmune diseases such as SLE, a breakdown of self-tolerance can occur due to environmental factors and genetic predisposition. Activation, proliferation, and antibody production are induced by external factors other than B cells, such as dendritic cells, helper T cells, and cytokines, and by the endogenous dysfunction of B cells (3). An abnormal balance of B-cell subsets, including the dysfunction of regulatory B cells and increased T-bet^+^CD11c^+^ B cells and plasmablasts, has been reported to be closely related to disease activity, autoantibody production, and organ damage in SLE (7–9).

**Figure 1:**
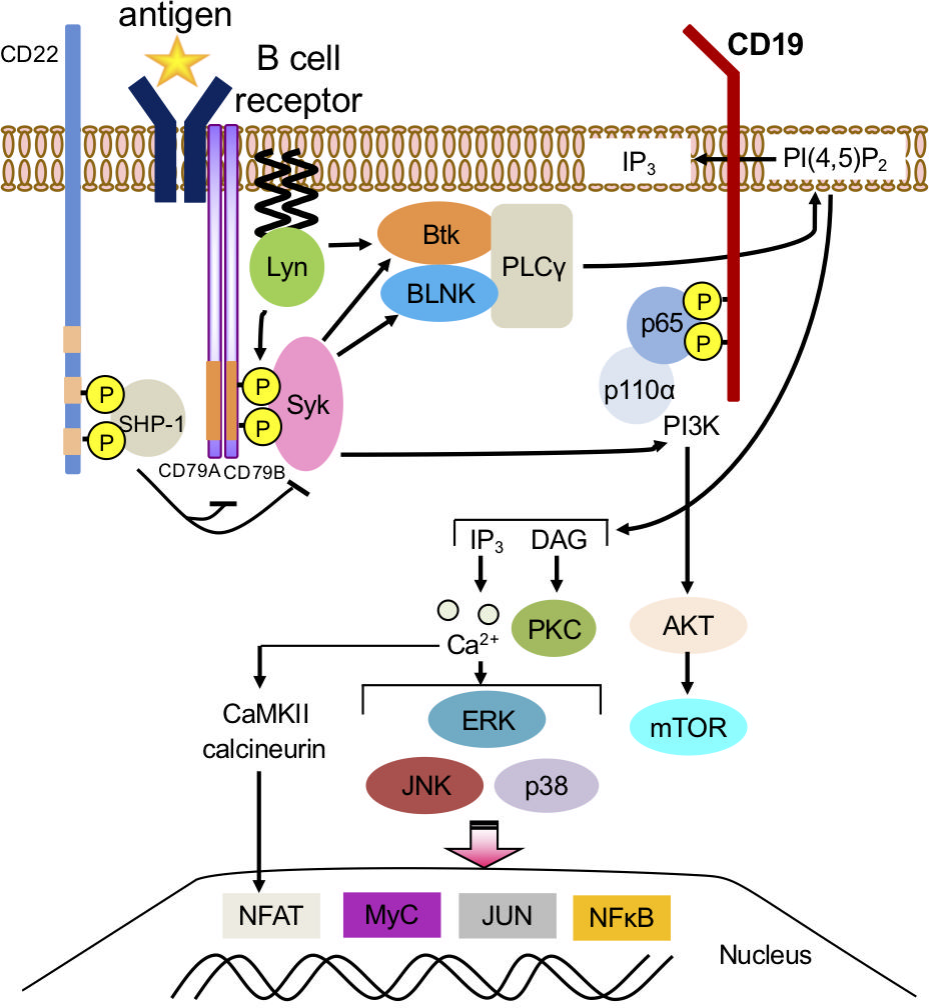
Downstream signaling pathway of B-cell receptors in B cells. Splenic tyrosine kinase (Syk) phosphorylates phospholipase C gamma 2 (PLCγ2). B-cell linker protein (BLNK) binds to Bruton’s tyrosine kinase (Btk) and PLCγ2, mediating PLCγ2 phosphorylation and activation, which cleaves phosphatidylinositol 4,5-bisphosphate (PI (4,5)P_2_) into inositol 1,4,5-trisphosphate (IP_3_) and diacylglycerol (DAG). IP_3_ binds to receptors on the endoplasmic reticulum. Extracellular signal-regulated kinase (ERK), c-Jun N-terminal kinase (JNK), p38 mitogen-activated protein kinase (MAPK) cascade translocates transcriptional factors such as NFκB and Nuclear factor of activated T-cells (NFAT) into the nucleus and initiates a B-cell response.

B cells are activated and differentiated by co-stimulatory molecular signals, including BCRs as the main signal and CD40/CD40L as a co-stimulatory signal, Toll-like receptors (TLR) 7,9, and cytokine signals such as IFN-α, IFN-γ, IL-2, and IL-4. B-cell activation is generally triggered by a ubiquitous pathway conformed by splenic tyrosine kinase (Syk) phosphorylates phospholipase C gamma 2 (PLCγ2). B-cell linker protein (BLNK) binds to Bruton’s tyrosine kinase (Btk) and PLCγ2, mediating PLCγ2 phosphorylation and activation. This cleaves phosphatidylinositol 4,5-bisphosphate (PI (4,5)P_2_) into inositol 1,4,5-trisphosphate (IP_3_) and diacylglycerol (DAG). The extracellular signal-regulated kinase (ERK), c-Jun N-terminal kinase (JNK), and p38 mitogen-activated protein kinase (MAPK) cascade translocate transcriptional factors into the nucleus (10–12) (**Figure 1**).

B cells from patients with SLE have heightened signaling responses, such as tyrosine phosphorylation and increased calcium flux, compared to healthy B cells (13) or in response to BCR cross-linking stimuli (14–16). Further, B cells from patients with SLE have increased levels of phosphorylated Lyn (17) and decreased PTEN activity, which inhibits BCR signaling (16). Syk is a 72 kDa non-receptor type protein tyrosine kinase (18) that is activated by multichain immune receptors, such as the Fc receptor (FcR) and BCR; it is widely expressed on immune cells, including macrophages, mast cells, neutrophils, and B cells (19, 20). Syk inhibitors have suppressed cutaneous and renal lesions in a mouse model of lupus (21, 22). We investigated the role of Syk in human B-cell activation *in vitro* and confirmed that Syk-mediated BCR signaling is important for the efficient induction of CD40 and TLR signaling (23). Moreover, we observed that Syk phosphorylation was increased in B cells from patients with SLE and positively correlated with disease activity (24).

Btk is another important intracellular kinase that regulates B-cell function. Mutations of Btk in humans cause X-linked agammaglobulinemia (XLA), a genetic disorder characterized by B-cell loss in peripheral blood and severely reduced Ig production (25). Transgenic mice overexpressing Btk have shown increased germinal centers and plasmablasts, while exhibiting autoantibody production and lupus-like pathology (26). The two interactions that occur, Btk-PH domain and PIP3 and Btk-SH2 domain and BLNK, mobilize Btk to the plasma membrane, activate phospholipase Cγ (PLCγ), and activate downstream signaling pathways following a calcium influx (27). We confirmed the importance of Btk in human B-cell activation and differentiation and observed increased levels of Btk phosphorylation in B cells from patients with SLE (24, 28). These data are consistent with the results of a GWAS in patients with SLE, in which nearly half of the patients showed genetic abnormalities related to B-cell signaling, such as PTPN22, BANK1, and BLK (29–31).

## Intracellular metabolism in survival, growth, activation, and differentiation

**Figure 2:**
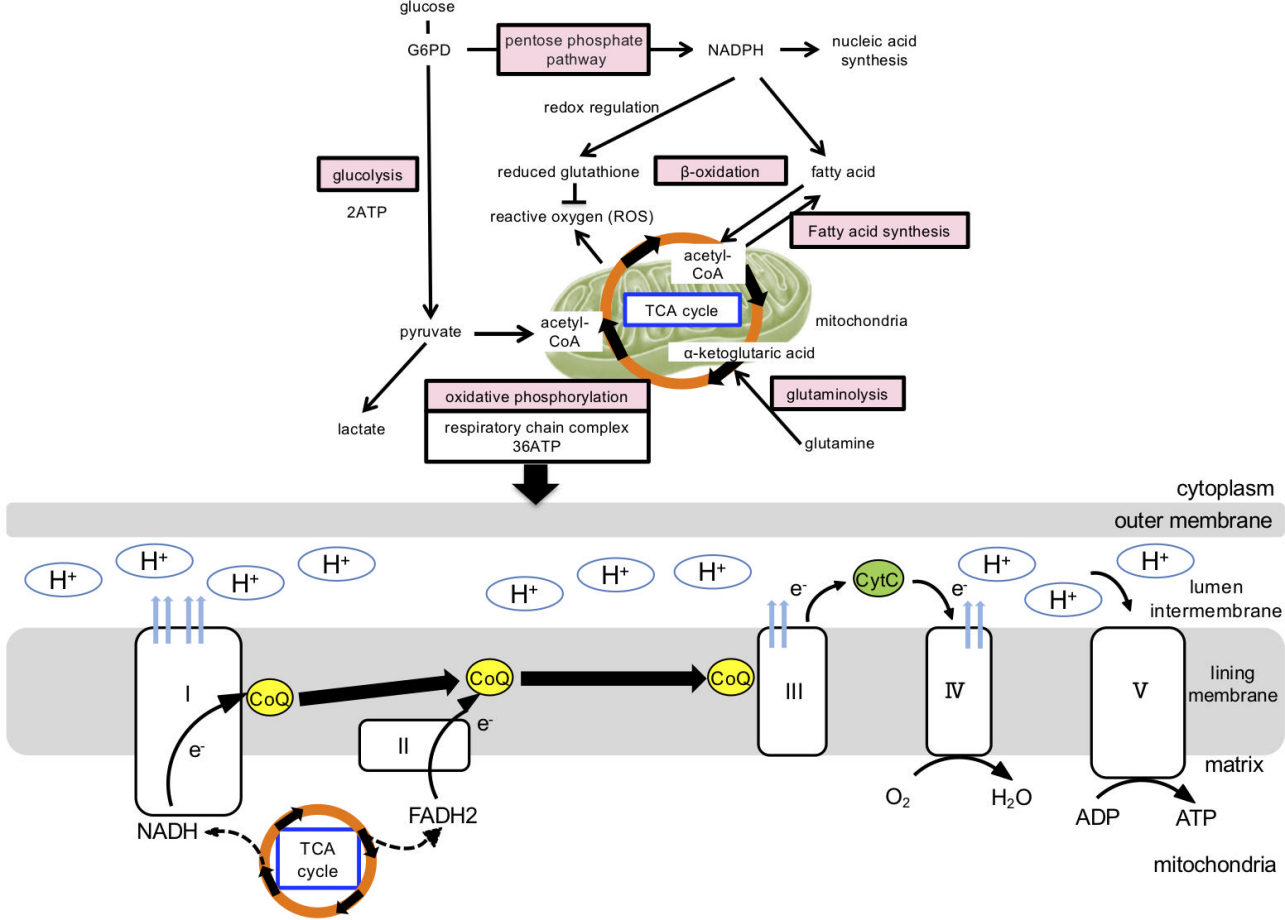
Cellular metabolic pathway and mitochondrial ATP production. Cellular energy production and generation of its constituent components involve the following metabolic pathways: glycolytic, oxidative phosphorylation, pentose phosphate circuit, fatty acid β-oxidation, fatty acid synthesis, and amino acid metabolism. In normal cells, oxidative phosphorylation is mainly utilized in the mitochondria, which produce the high-energy compound ATP most efficiently, and the metabolisms of carbohydrates, lipids, and amino acids converge during this reaction. Glucose-derived pyruvate and fatty acids are converted into acetyl-coenzyme A (acetyl-CoA), which enters the TCA cycle. Glutamate is directly converted to α-ketoglutarate, which enters the TCA cycle as an important fuel. In the electron transfer system, ATP is synthesized via oxidative phosphorylation. Oxidative phosphorylation is a chemical reaction that utilizes the hydrogen of NADH+H+ and FADH2 generated in the citric acid circuit and is coupled with the mitochondrial electron transfer system. H is transported through the electron transfer system to the mitochondrial intermembrane lumen, increasing hydrogen ion concentration, thus, forming a concentration gradient. ATP synthase is present in the inner mitochondrial membrane and serves as a pathway for hydrogen ions to flow into the mitochondrial matrix, rotate some molecules, and use their energy to synthesize ATP from ADP. G6PD, glucose-6-phosphate dehydrogenase; NADH, Nicotinamide adenine dinucleotide phosphate; NADPH, reduced nicotinamide adenine dinucleotide phosphate; TCA, tricarboxylic acid; FADH2, reduced flavin adenine dinucleotide; CoQ, CoenzymeQ; ADP, adenosine diphosphate; ATP, adenosine triphosphate.

The production of energy and key components for cell survival and growth involves six main metabolic pathways: glycolysis, oxidative phosphorylation, the pentose phosphate circuit, fatty acid synthesis, fatty acid β-oxidation, and amino acid metabolism (glutaminolysis) (**Figure 2**). These are regulated by HIF1α, c-myc, mTOR, AMPK, and other metabolic regulators (32). In normal cells, a series of ATP-synthesis reactions occur in concert with the electron transfer system (i.e., oxidative phosphorylation in the mitochondria), which most efficiently produces the high-energy compound ATP. The metabolisms of carbohydrates, lipids, and amino acids converge during this reaction. Glucose-derived pyruvate and fatty acids are converted into acetyl-coenzyme A (acetyl-CoA), which enters the TCA cycle. Glutamate is directly converted to α-ketoglutarate, an important fuel and intermediate in the TCA cycle. NADH and FADH2, key products of the TCA cycle, transfer electrons to the electron transport system, supporting oxidative phosphorylation and, thus, the highly efficient ATP production (36 ATP molecules produced per 1 molecule of glucose) (**Figure 2**). Lactate production via the glycolytic system has been observed under anaerobic conditions (33).

In contrast, activated immune cells exhibit characteristic metabolic alterations that require the biosynthesis of large amounts of nucleic acids, lipids, and other bioconstituents through metabolic transformations. First, ATP is produced using a glycolytic system, which is less efficient than oxidative phosphorylation (two ATP molecules are produced per one molecule of glucose). Cell proliferation is possible even under hypoxic conditions, as oxygen is not required. Second, NADPH, maintained by the pentose phosphate pathway or glutaminolysis (glutamine degradation) following glycolysis, is used for redox regulation (reduced glutathione production) and fatty and nucleic acid synthesis. Reduced glutathione suppresses ROS production in mitochondria and maintains the redox balance (34).

## Role of the PI3K-Akt-mTORC pathway on differentiation and class switching of B cells

The mechanism of intracellular metabolism in B-cell activation has been mainly reported in mouse models: enhanced PI3K-Akt-mTORC signaling, glycolysis, and oxidative phosphorylation induce *de novo* lipid synthesis, which is important for B-cell proliferation and growth (35). The mTORC1 and mTORC2 complexes are the two serine-threonine kinases inhibited by rapamycin treatment (36–38). Overexpression of mTORC1 promotes plasma cell differentiation, whereas rapamycin inhibits B-cell proliferation and survival (39, 40). Rictor deletion, which encodes an essential subunit of mTORC2, suppresses B-cell proliferation by decreasing cell cycle and survival signals (41). Deletion of SIN1—an essential subunit of mTORC2—with Cd19-Cre inhibits proliferation and antibody production (42). However, a complex regulatory mechanism has been postulated for PI3K-Akt-mTORC signaling in B-cell class-switching. Increased PI3K activity and selective inhibition of the p110δ catalytic isoform indicated that PI3K suppresses class switching (43–46). In contrast, inhibition of AKT completely canceled class-switching suppression by PI3K activation (45). mTORC1 and mTORC2 are promoters and inhibitors of class switching, respectively (47). AKT may be predominantly regulated by mTORC2 to inhibit class switching, independent of mTORC1 (48). The B-cell activating factor (BAFF) and its homolog APRIL support B-cell differentiation and plasma cell survival while regulating immunoglobulin class switching (49, 50). *In vitro* studies have shown that rapamycin inhibits BAFF-mediated proliferation and survival signals (40).

## Plasmablast differentiation via essential amino acids-mTOR pathway in B cells in SLE

mTOR is a serine/threonine kinase conserved from yeast to humans that senses various signals inside and outside the cell, including growth factors, amino acids, and stress, and regulates cell growth, metabolism, and survival (51). In the B cells of a mouse lupus model, mTORC1 was activated, and the lupus-like pathology was ameliorated by rapamycin (39). We confirmed that p-mTOR expression levels were elevated in B cells from patients with SLE compared to those from healthy controls and correlated with plasmablast percentages and disease activity (52). We investigated the effects of amino acids on B-cell differentiation and function via mTOR, their regulatory mechanisms, and their relevance in SLE pathogenesis (53).

CD19-positive cells were isolated from the peripheral blood of healthy subjects and stimulated *in vitro* with BCR cross-linking, CpG, a TLR (Toll-like receptor) 9 ligand, and IFN-α, which induced CD27highCD38high plasmablasts. The uptake of essential amino acids—leucine and methionine—was enhanced. Plasmablast differentiation was partially inhibited by leucine deficiency and completely suppressed by methionine deficiency. These results suggest that these essential amino acids are important for plasmablast differentiation (53). The discovery of three amino acid sensors–SLC38A9, Sestrin1/2, and CASTOR1–revealed a partial sensing mechanism for leucine and arginine (54). dSestrin has been shown to act as a methionine sensor under certain conditions (55). We observed that Syk, PLC-γ phosphorylation, and TRAF6 expression were suppressed under methionine deficiency; however, they were unchanged under leucine deficiency. In contrast, AKT and S6 phosphorylation were downregulated under both deficiencies, whereas c-myc and HIF-1α expression showed no effect. Methionine deficiency suppressed aerobic glycolysis, fatty acid synthesis, ROS production, and glutamate uptake (53).

BACH2 is important in antigen-stimulated B cell and germinal center B-cell differentiation, class switching, and somatic hypermutation (56). BACH2 suppresses the expression of Prdm1, a gene encoding BLIMP1. Downregulation of BACH2 expression and induction of BLIMP1 expression promote plasmablast differentiation (57). We observed that BACH2 expression was downregulated in CD19-positive cells in patients with SLE compared to that in cells from healthy controls. *In vitro* stimulation of healthy human CD19-positive cells with BCR cross-linking, CpG, and IFN-α decreased BACH2 expression and enhanced IRF4, PRDM1, and XBP1 expressions, which was disrupted under methionine deficiency (53).

**Figure 3:**
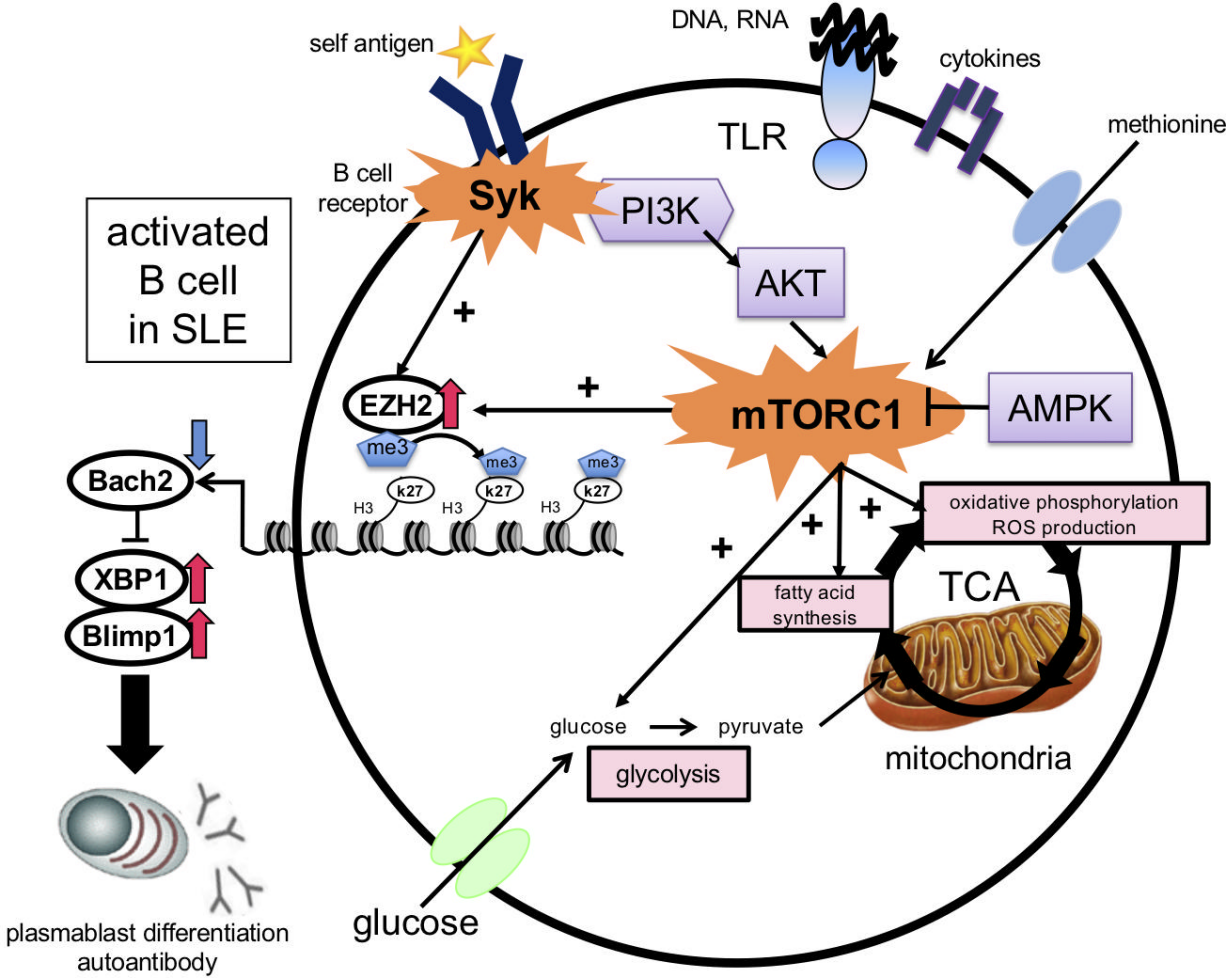
Induction of plasmablast differentiation mechanism via the essential amino acid methionine in B cells in SLE. Stimulation with BCR cross-linking, Toll-like receptors, and cytokines in B cells in the presence of methionine enhances glycolysis, fatty acid synthesis, and ROS production. EZH2 induces H3K27me3 at the BACH2 locus, which suppresses BACH2 expression and promotes plasmablast differentiation by inducing BLIMP1 and XBP1 expression. BCR, B cell receptor; ROS, reactive oxygen species; EZH2, enhancer of zeste homolog 2; TLR, Toll-like receptor; PI3K, phosphatidylinositol-3 kinase; AMPK, AMP-activated protein kinase; BACH2, BTB, and CNC homolog 2; BLIMP-1, B lymphocyte-induced maturation protein-1; XBP-1, X-box binding protein 1.

Methionine is the precursor of the methyl group donor S-adenosylmethionine (SAM), which is utilized by all histone methyltransferases, including EZH2, a core subunit of the polycomb repressive complex2 (PRC2) protein complex (58). EZH2 methylates H3K27 and represses gene expression; H3K27me3 is located near the promoters and transcribed regions of repressor genes and at the promoters of activated repressor genes (59). BCR cross-linking and CpG and IFN-α stimulation induce EZH2 expression. EZH2 binds to the BACH2 promoter region and induces H3K27me3 expression. The level of EZH2 in B cells is positively correlated with the anti-dsDNA antibody titer and the disease activity indices SLEDAI and BILAG. This suggests that Syk and mTORC1 activation synergistically induce EZH2 expression in B cells in the presence of methionine, suppress BACH2 expression via epigenomic modifications, and induce plasmablast differentiation in SLE (53) (**Figure 3**).

## Role of mitochondria and their abnormalities in immune cells in SLE

Reportedly, mTOR induces mitochondrial activation and proliferation, promoting anabolic processes such as mRNA translation, glycolysis, and fatty acid synthesis (60). Most of the proteins involved in mitochondrial energy production are encoded in the nucleus. mTORC1 indirectly regulates energy production by regulating the translation of nuclear-encoded mitochondrial proteins (61).

Mitochondria have many functions in cellular homeostasis, and their disruption can lead to disease development. The most characteristic function of mitochondria is the regulation of cellular metabolism, in which NADH and FADH2 are produced via the cytosolic and mitochondrial pathways. The electron transport chain uses NADH and FADH2 to pump protons into the intermembrane lumen, forming an electrochemical gradient through which ATP is synthesized from ADP by ATP synthase (62). Electrons exit the electron transfer system and react directly with oxygen dioxide to form mitochondrial reactive oxygen species (mtROS), which contribute to inflammation (63–66). Mitochondria possess their circular genome (mtDNA), which has been investigated for its possible involvement in rheumatic diseases. mtROS can induce oxidative damage to nuclear DNA/mtDNA, post-translational oxidation of local proteins, and lipid peroxidation (67–69). Compared to nuclear DNA, mtDNA is ten times more susceptible to damage by mutagenic agents such as ROS. Mitochondria are a source of ROS; they do not contain DNA-protecting proteins such as histones, rendering their DNA repair system insufficient (70).

In patients with SLE, mitochondrial abnormalities have been reported to be involved in disease pathogenesis in immune cells other than B cells (71). Mitochondrial dysfunction may be caused by mitochondrial genomic mutations or chronic exposure to inflammatory cytokines, including type I IFNs. CD8+ T cells from patients who have SLE with a high type I IFN gene signature are more susceptible to spontaneous cell death than those from healthy individuals or patients lacking this signature. These cells have various mitochondrial defects, including increased mitochondrial mass, mitochondrial hyperpolarization, and decreased respiratory capacity (72). Oxidized mtDNA released from the neutrophils of patients with SLE induces type I IFN production by plasmacytoid dendritic cells (pDCs) (73). In addition, pDCs are activated by oxidized mtDNA-induced TH10 (CXCR5^-^CXCR3^+^PD1^hi^CD4^+^ cells) (74). Neutrophils activated by immune complexes containing U1 small nuclear ribonucleoproteins (SnRNPs) produce mtROS and induce NET formation and release (64). NETs contain highly oxidized mtDNA and induce cGAS-STING-dependent type 1 IFN production. However, the role of mitochondria in SLE B cells and their involvement in cell differentiation remain unclear.

## Mitochondrial hyper function and glutaminolysis induce plasmablast differentiation in SLE

To confirm the presence of mitochondrial abnormalities in B cells from patients with SLE and their involvement in SLE pathogenesis, we compared patients with SLE (n = 41) with healthy controls (n = 26) (75). 3,3’-Dihexyloxacarbocyanine iodide (DiOc6) is a cell-permeant, fluorescent green, lipophilic dye that indicates mitochondrial membrane hyperpolarization. The expression was significantly higher in B cells from patients with SLE than in healthy control cells. Furthermore, the expression level of DiOc6 in B cells was significantly positively correlated with the percentage of plasmablasts among CD19^+^ cells and SLEDAI (75). Recently, a multi-omics analysis identifying mRNA expression profiles and gene polymorphisms in patients with SLE and healthy controls has been reported. This study showed an association between oxidative phosphorylation/mitochondrial dysfunction in transcripts, genes, and the epigenome, and type 1 IFN and oxidative phosphorylation signatures in memory B cells of patients with SLE (76), supporting our results.

Nutrients such as glucose and glutamine are required for mitochondrial function. Stimulation of CD19^+^ cells with CpG and IFNα increased DiOc6 expression, the oxygen consumption rate (OCR), and the ^14^C-glutamine uptake. This, in turn, induced plasmablast differentiation, which was suppressed under glutamine deficiency compared to normal conditions (75). A previous report showed that l-glutamine was important for B-cell differentiation (77) and glutamine uptake was enhanced when mouse B cells were activated (78), supporting our results.

Glutamine is involved in metabolic regulation and is a key amino acid in protein synthesis. Therefore, we speculated that glutamine deficiency may have affected metabolism, plasmablast differentiation, and antibody production. Therefore, we limited our study to glutamine degradation (i.e., glutaminolysis). BPTES, a glutaminase inhibitor that selectively inhibits glutaminolysis, suppressed ROS production and OCR but did not affect ECAR (75). Mitochondria consume approximately 95% of the oxygen *in vivo*, 1–3% of which is converted to ROS (79). The functional significance of ROS in B cells has been reported as follows: ROS production by B-cell activation enhances BCR and other signaling pathways, for example, by inhibiting SHP-1 and other signaling pathways (80, 81). In addition, increased ROS levels are associated with plasmablast differentiation induced by senescent mitochondrial stagnation (82).

Recently, glutaminolysis was abnormally enhanced in peripheral blood lymphocytes from patients with SLE and lupus model mice. Suppression of glutaminolysis reduced the number of helper T cells and activated B cells. In contrast, the activated mTOR/p70S6k/4EBP1 and NLRP3/capsase-1/IL1-β pathways were reportedly suppressed (83). Furthermore, BPTES ameliorated SLE pathology by regulating Th17 cells in MRL/Lpr mice (80). Thus, the glutaminolysis of B and T cells may play an important role in SLE pathogenesis.

**Figure 4:**
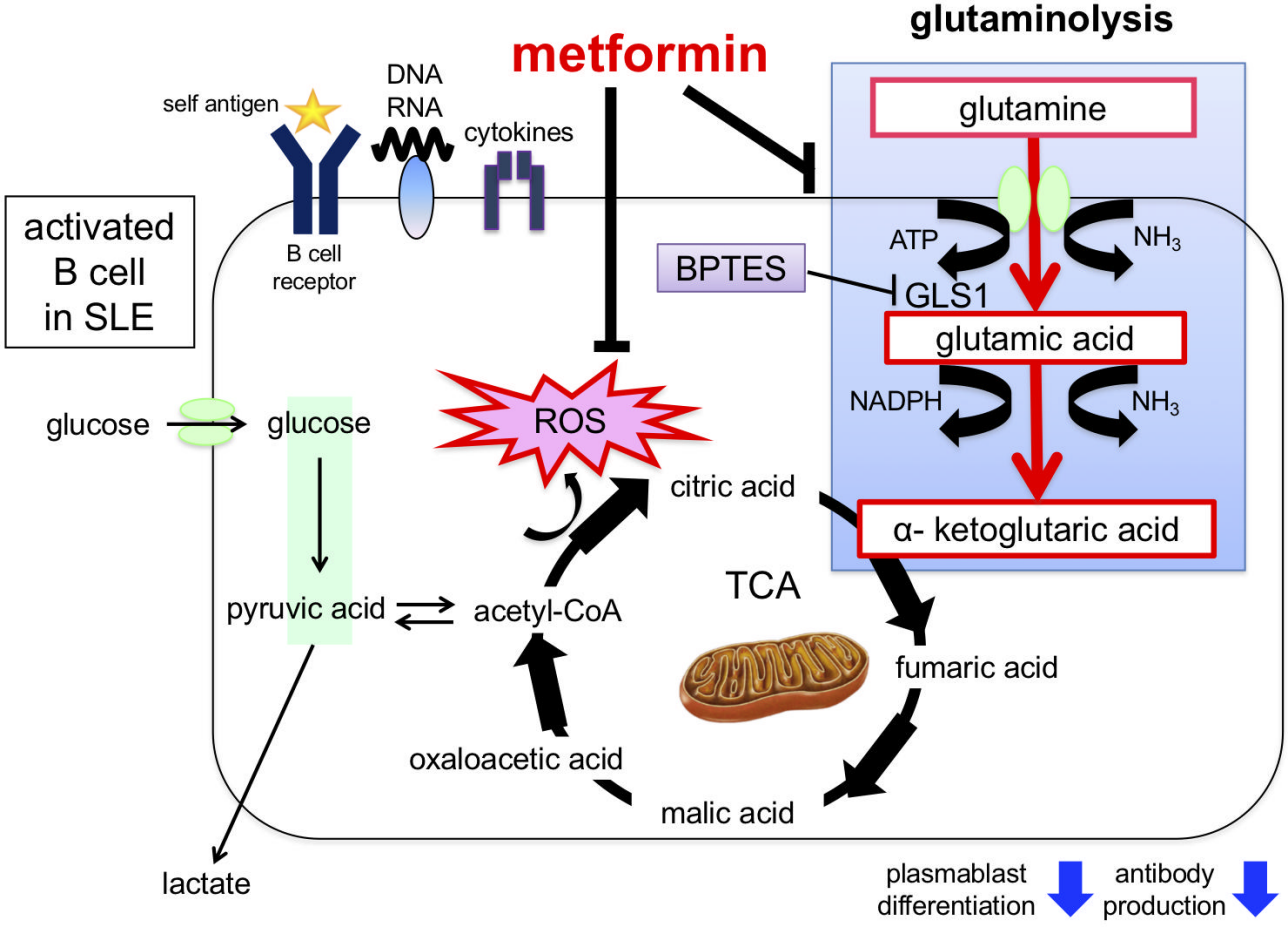
Induction of plasmablast differentiation mechanism via glutaminolysis and enhancement of mitochondrial function in B cells from patients with SLE. Stimulation by BCR cross-linking, Toll-like receptors, and cytokines in B cells promotes glutamine uptake and mitochondrial function (e.g., ROS production), which induces plasmablast differentiation. Glutaminolysis involves a series of biochemical reactions that break down the amino acid glutamine into glutamic acid, aspartic acid, CO_2_, pyruvic acid, lactic acid, alanine, and citric acid. Plasmablast differentiation is inhibited by adding the glutaminase inhibitor BPTES. In addition, metformin inhibits glutamine uptake and ROS production and suppresses plasmablast differentiation. ROS, reactive oxygen species; BPTES, glutaminase inhibitors; GLUT1, glucose transporter 1; GDH, glutamate dehydrogenase; GLS, glutaminase.

Metformin suppresses ROS generation, activates AMPK, and indirectly inhibits mTORC1 activation by inhibiting reverse electron flux transfer in mitochondrial respiratory chain complex I (84, 85). In mouse B cells, metformin has been reported to increase AMPK and suppress mTOR-STAT3 signaling, inhibiting plasma cell differentiation (86). We observed that the enhanced OCR, DiOc6 expression, ROS production, ATP production, glutamine uptake, and plasmablast differentiation induced by CpG and IFNα were inhibited by metformin. Furthermore, plasmablast differentiation and antibody production were significantly suppressed by the addition of metformin to SLE B cells (75). These results indicate that metformin regulates excessive oxidative phosphorylation via glutamine metabolism and suppresses plasmablast differentiation and antibody production (**Figure 4**).

## Immunometabolic dysregulation of CD11c+ T-bet+ B cells in patients with SLE

The details of the differentiation stage in which SLE B cell defects originate have been unknown; however, advances in analytical methods have gradually revealed their origin. A genome-wide analysis showed that resting naïve B cells had already undergone epigenomic alterations such as DNA methylation and chromatin accessibility (87).

In SLE, the percentage of peripheral CD11c^+^T-bet^+^ B cells is closely associated with pathological conditions such as the production of anti-ds-DNA/SM/RNP and other antibodies, disease activity, and renal damage (9). CD11c^+^T-bet^+^ B cells are similar to mouse ABCs (age- and/or autoimmunity-associated B cells) and were detected in the fraction of activated naïve B cells (acN) and IgD^-^CD27^-^CD11c^+^T-bet^+^CXCR5^-^DN2 cells. Furthermore, single-cell RNA-seq analysis of renal tissues obtained from healthy subjects and patients with lupus nephritis confirmed the presence of activated naïve B cells (CD11c^+^T-bet^+^ B cells) mentioned above in renal tissues. This was consistent with their association with renal injury in peripheral blood (88).

Wu et al. identified the role of mTOCR1 and its gene expression profile in lupus-associated atypical memory B cells (i.e., CD11c^+^T-bet^+^ B cells). They reported that B-cell signaling, the mTORC1 pathway, lipid/carbohydrate metabolism, and endocytosis pathways were abnormally activated and functionally dysregulated. Further, differentiation of lupus-associated atypical memory B cells was inhibited by rapamycin (89). However, the mechanisms underlying the differentiation, function, and metabolic control of CD11c^+^T-bet^+^ B cells remain unclear.

## Immunometabolic regulation in regulatory B cells (Breg) and its association with autoimmune diseases

In recent years, Breg dysfunction in patients with SLE has received increasing attention. In experimental autoimmune encephalomyelitis (EAE), variations in disease onset, severity, and degree of recovery have been observed in B cell-deficient mice (90). Additionally, in the 2000s, it was shown that Breg cells are special B cells that produce IL-10 and have immunosuppressive properties. Subsequent studies in mice showed that CD1d^hi^IgM^hi^CD21^hi^CD23^hi^ and CD5^+^CD1d^hi^ (B10 cells) are IL-10-producing B cells. In humans, Breg surface antigens include CD24^hi^CD38^hi^, CD24^hi^CD27^+^, CD38^+^CD1d^+^IgM^+^CD147^+^, CD25^hi^CD71^hi^CD73^hi^, CD27^int^CD38^hi^, CD39^+^CD73^+^, and CD19^+^TIM1^+^ cells (91). Matsumoto et al. reported that B cell subsets expressing IL-10 *in vitro* are plasmablasts that differentiate into lymph nodes in reporter mice (92).

Several reports describe the mechanisms underlying Breg differentiation and functional induction. Storage-operated calcium (SOC) influx is involved in IL-10 production and triggered by calcium depletion from the ER, which is dependent on stromal interaction molecule (STIM) 1 and STIM2 sensors. B-cell-specific deletion of STIM1 and STIM2 reduces IL-10 production in mice and exacerbates autoimmune diseases. In other words, STIM-dependent SOC influx upon BCR stimulation is an important signal for Breg cell differentiation, suppressing autoimmunity (93). The aryl hydrocarbon receptor (AhR) is an important regulator of innate and adaptive immune cell development and function (94). Recently, AhR was reported to regulate the differentiation and function of IL-10-producing CD21^hi^CD24^hi^ Breg cells in both mice and humans (95). In AhR-deficient mice, IL-10-producing Breg and regulatory T (Treg) cells are substantially reduced, and arthritis is exacerbated. The levels of butyrate, a short-chain fatty acid (SCFA), were decreased in arthritic mice and patients with rheumatoid arthritis (RA). Butyric acid supplementation induced the production of two AhR ligands associated with Trp metabolism, 5-hydroxyindole-3-acetic acid and kynurenic acid, and increased IL-10 production in Breg cells. Further, AhR-dependent IL-10 Breg induction in B cells inhibits GC and plasmablast differentiation and regulates arthritis (96).

Blair et al. reported that CD19^+^CD24^hi^CD38^hi^ cells from healthy individuals suppressed Th1 cell differentiation via CD40-stimulated IL-10 production. However, the same cells from patients with SLE did not respond to CD40 stimulation, lacked IL-10 production, and could not suppress Th1 cell differentiation (7). In addition, they reported that CD24hiCD38hi immature B cells from healthy individuals co-cultured with pDCs+CpG A (TLR9 ligand) strongly produced IL-10 and suppressed IFN-α production by pDCs. Contrastingly, CD24^hi^CD38^hi^ immature B cells from patients with SLE showed reduced IL -10 production, indicating impaired suppression of IFN-α production by pDCs (8).

There are few reports on the immunometabolism of Breg cells. Stimulation of naïve B cells with anti-CD40, anti-IgM, and/or lipopolysaccharide (LPS) induces IL-10 production by B cells and increases glycolytic flux (77). HIF1α regulates glycolysis and glycolytic gene expression and binds to a putative hypoxia response element within the Il10 locus, in conjunction with pSTAT3, to transcriptionally regulate IL-10 production in B10 cells (97).

## Immunometabolic mechanisms in B cells and future prospects

Various agents are currently undergoing clinical trials for SLE. These are telitacicept (BlyS and APRIL inhibitors), anti-CD20 antibodies (rituximab, obinutuzumab), anti-CD40L antibody (dapirolizumab pegol), JAK inhibitors (JAK1 selective inhibitor upadacitinib, Tyk2 selective inhibitor deucravacitinib), low-volume soluble IL-2 (ILT-101), anti-IL-17A antibody secukinumab, anti-IL-23 antibody guselkumab, and anti-BDCA2 antibody BIIB059, among others (98). Recently, the usefulness of CAR-T therapy in patients with SLE has been reported (99). However, a further understanding of SLE pathogenesis and the exploration of novel therapeutic strategies are essential. Targeting immunometabolism is an attractive therapeutic strategy. Metabolic modulators such as sirolimus, rapamycin, and metformin have shown efficacy and tolerability in mouse model and clinical trials (100–106). However, the efficacy and safety of these drugs, alone or in combination with other DMARDs, require further research. Furthermore, clarification of the immune-metabolic regulatory mechanisms specific to each immune cell in autoimmune diseases, such as SLE, is important for the development of novel therapies to combat them.

## Author contributions

SI and MS-H wrote this manuscript. YT has reviewed and edited the manuscript. All authors contributed to the article and approved the submitted version.
